# Privacy-preserving *k*-NN interpolation over two encrypted databases

**DOI:** 10.7717/peerj-cs.965

**Published:** 2022-05-31

**Authors:** Murat Osmanoglu, Salih Demir, Bulent Tugrul

**Affiliations:** Department of Computer Engineering, Ankara University, Ankara, Turkey

**Keywords:** Big data, Cloud computing, Interpolation, *k*-nearest neighbour

## Abstract

Cloud computing enables users to outsource their databases and the computing functionalities to a cloud service provider to avoid the cost of maintaining a private storage and computational requirements. It also provides universal access to data, applications, and services without location dependency. While cloud computing provides many benefits, it possesses a number of security and privacy concerns. Outsourcing data to a cloud service provider in encrypted form may help to overcome these concerns. However, dealing with the encrypted data makes it difficult for the cloud service providers to perform some operations over the data that will especially be required in query processing tasks. Among the techniques employed in query processing task, the *k*-nearest neighbor method draws attention due to its simplicity and efficiency, particularly on massive data sets. A number of *k*-nearest neighbor algorithms for query processing task on a single encrypted database have been proposed. However, the performance of *k*-nearest neighbor algorithms on a single database may create accuracy and reliability problems. It is a fact that collaboration among different cloud service providers yields more accurate and more reliable results in query processing. By considering this fact, we focus on the *k*-nearest neighbor (*k*-NN) problem over two encrypted databases. We introduce a secure two-party *k*-NN interpolation protocol that enables a query owner to extract the interpolation of the *k*-nearest neighbors of a query point from two different databases outsourced to two different cloud service providers. We also show that our protocol protects the confidentiality of the data and the query point, and hides data access patterns. Furthermore, we conducted a number of experiment to demonstrate the efficiency of our protocol. The results show that the running time of our protocol is linearly dependent on both the number of nearest neighbours and data size.

## Introduction

Due to its low cost, scalability and reliability, cloud computing has increased its reputation in both the business and scientific communities. In addition to the benefits, it introduces new concerns that need to be addressed carefully (Krutz & Vines, 2010). One of the emerging issues in cloud computing is extracting knowledge from sensitive data while protecting the privacy of data owners, which is called privacy-preserving data mining (Agrawal & Srikant, 2000; Vaidya & Clifton, 2004). A privacy-preserving data mining method aims to provide data privacy using either data perturbation or cryptographic methods. Data perturbation-based models struggle with data quality issues, *i.e*. the valuable statistical information might be dissolved. This may yield less accurate and less reliable results. On the other hand, cryptographic-based models achieves the privacy of data owners through the encryption of data before outsourcing it to the cloud. However, this presents challenges of performing required operations over the encrypted data.

In addition to these facts, collaboration among different cloud service providers may also help them to create more accurate and reliable results in a privacy-preserving data mining method, *i.e*. more clouds can discover more knowledge than they can uncover on their own when they combine their data (Demir & Tugrul, 2018). There are some studies that propose privacy-preserving solutions for horizontally-partitioned databases to increase the total number of data samples with the goal of creating more accurate data mining models (Inan et al., 2007). In some cases, vertically-partitioned database solutions can be preferred to increase the number of attributes for the same instances (Skillicorn & McConnell, 2008). Institutions such as hospitals operating in different parts of a country may prefer the first choice. On the other hand, institutions such as banks and insurance companies may aggregate their data using the second choice.

In this study, we will examine the *k*-NN interpolation method that preserves the confidentiality of two different databases stored by two different cloud service providers. *k*-NN, categorized as a lazy learner, is a non-parametric method used for classification, clustering and interpolation which utilizes the idea that neighboring objects possess or display similar characteristics. Complex interpolation methods such as Kriging involve advanced operations and thus pose a great challenge to cloud computing. In addition, the high time requirements of such methods make them unsuitable in some scenarios such as healthcare applications. On the contrary, the simplicity and interpretability of the *k*-NN method make it an efficient tool for query processing tasks.

### Our contribution

In this article, we introduce an efficient secure two-party *k*-NN (STPkNN) interpolation protocol that enables two different data owners to outsource their databases together with the query processing service to the cloud, and allows a query owner to extract the interpolation of the *k*-nearest neighbors of a query point from the encrypted databases. Our protocol preserves the confidentiality of data, assures the privacy of user’s query point, and hides data access patterns.

The STPkNN protocol can be considered as an extension of the protocol SkNN_*m*_ proposed in Elmehdwi, Samanthula & Jiang (2014), that enables a query owner to retrieve the *k*-nearest neighbors of a query point from a single encrypted database, to two-cloud settings. Briefly, the SkNN_*m*_ protocol calculates the *k*-nearest neighbors in an iterative way by performing the following steps *k* times: (i) it finds the minimum of the Euclidean distances between the data records and the query point, (ii) it calculates the one of the nearest neighbors that corresponds to the index of the minimum distance, and excludes the corresponding distance from the Euclidean distances. On the other hand, in two-cloud settings, the clouds have to share their local minimums of the Euclidean distances to decide on the global minimum that corresponds the index of the nearest neighbor of two databases at the moment, and remove that record from further iterations. However, it is not trivial to achieve this without revealing which data record corresponds to global minimum to any cloud.

To this aim, we first propose two new security primitives, the Secure Transformation (ST) protocol and the Secure Bit-AND-OR (SBAOR) protocol that enable the clouds to decide on the global minimum and exclude it from the further calculations without revealing data access pattern to any cloud. We show that both protocols protect the confidentiality of the input values which will be in encrypted form, *i.e*. no information about the input values is leaked to any party during the protocols, and the output is only revealed to one of the parties in the protocols. Briefly, the ST protocol allows the servers to securely transform the encryption of a record under a public key to an encryption of same record under another public key. On the other hand, for given the encryptions of two bit vectors *x* and *y*, the SBAOR protocol enables the servers to securely compute the negation of the logical disjunction of all bitwise multiplications *x*_*i*_ · *y*_*i*_ in encrypted form without revealing the bit vectors to any party.

By employing the ST and SBAOR protocols together with the other existing security protocols, we build our main protocol STPkNN that enables a query owner (QO) to extract the interpolation of the *k*-nearest neighbors of a query point chosen by QO from two different databases outsourced to two different cloud service providers. In the protocol, data owners encrypt their data before outsourcing them to the cloud service providers, and they do not participate in the STPkNN protocol. Thus, no information about the data is leaked to the cloud service providers during the protocol. Besides, our protocol guarantees that any record from both databases or any intermediate result generated in the protocol is not leaked to the cloud service providers. Also, it hides the data access pattern from both data owners and cloud service providers, *i.e*. the protocol does not reveal the information of which data records were used to produce the interpolation of *k*-nearest neighbors to any cloud service provider. On the other hand, the STPkNN protocol outputs the interpolation of *k*-nearest neighbors only to the query owner, and the query owner gets no information other than the interpolation.

We also conduct various experiments on two real-world datasets from the UCI machine learning repository, the cervical cancer (risk factors) dataset and the default of credit card clients dataset, to show the practicability of our protocol in real world scenarios. The experimental evaluation presents that our protocol scales well for the large datasets.

### Related works

Due to its usefulness in many application scenarios such as classification, similarity search, and collaborative filtering, the problem of computing the *k*-nearest neighbors of a query point has been gained a lot of attention in recent years. The early studies mostly focused on how to implement a secure *k*-NN method between data owner and clients without using cloud systems. Shaneck, Kim & Kumar (2009) proposed a privacy-preserving protocol that employs secure multiparty computation to compute *k*-NN in horizontally partitioned databases. Besides, they also showed how their protocol can be efficiently used in different application such as outlier detection, classification, and clustering problems. Moreover, Qi & Atallah (2008) proposed a provable secure protocol for the single-step *k*-NN search problem that enjoys linear computation and communication complexity. Vaidya & Clifton (2005) introduced a privacy-preserving algorithm that performs top-*k* queries in vertically partitioned data. Additionally, Kantarcoğlu & Clifton (2004) proposed a method that privately calculates the *k*-NN classification over horizontally partitioned data in the distributed database model. Note that all of the above methods require the data owners to perform the necessary calculations to generate the result, and to return it directly to the query users. However, in our model, the data is outsourced to the cloud in encrypted form instead of being kept by the data owners. All of the computation required to process *k*-NN queries are performed by the cloud.

The recent studies have mostly focused on solutions in cloud computing settings. Wong et al. (2009) proposed an asymmetric scalar-product-preserving encryption (ASPE) scheme that can be employed to construct a secure *k*-NN protocol. The protocol proposed in Wong et al. (2009) uses a distance comparison function instead of an exact distance calculation. However, the secret key in the protocol should be disclosed to the query users. Zhu, Huang & Takagi (2016) introduced a secure protocol that achieves *k*-NN query processing on encrypted data without totally revealing the data owner’s secret key to the query user. However, their scheme requires data owners to be involved in the encryption of query points. Hu et al. (2011) proposed a secure traversal framework that can used, together with privacy homomorphism, to achieve secure *k*-NN query processing protocol. Cheng et al. (2015) proposed a privacy-preserving protocol that employs an encrypted hierarchical index tree to perform *k*-NN queries over spatial data outsourced to cloud in encrypted form. All three protocols (Hu et al., 2011; Zhu, Huang & Takagi, 2016; Cheng et al., 2015) leak data access pattern to the cloud. On the other hand, Kesarwani et al. (2018) proposed a secure *k*-NN query processing protocol over encrypted data by utilizing a leveled fully homomorphic encryption scheme. Wu et al. (2019) introduced a privacy preserving *k*-NN classification scheme over the encrypted cloud database that is secure against known-plaintext attack. Besides, Lei et al. (2020) shed light on the connection between a secure *k*-NN query processing scheme and a secure range query scheme. Based on this connection, they utilize a secure range query scheme together with a data structure named as random Bloom filter to build a secure *k*-NN query processing scheme. All three protocols (Kesarwani et al., 2018; Wu et al., 2019; Lei et al., 2020) hide data access pattern as well as preserving the data privacy and query privacy. However, they require the decryption keys to be given the query users. However, in our model, the decryption keys are not shared with the query users.

On the other hand, Elmehdwi, Samanthula & Jiang (2014) tackled with the same problem using homomorphic encryption method. In addition to ensuring the confidentiality of data owners and clients, the protocol proposed in Elmehdwi, Samanthula & Jiang (2014) also achieves to hide data access patterns from the clouds. Moreover, Xu et al. (2017) proposed an efficient secure *k*-NN protocol which achieves sublinear computational complexity. Similar to Elmehdwi, Samanthula & Jiang (2014), their protocol also achieves hiding of data access patterns using garbled circuits to simulate Oblivious RAM. Furthermore, Guo & Sun (2020) adopted the data structure R-tree to build an efficient *k*-NN scheme that requires only two rounds of interactions between the client and cloud servers to generate the result. They also utilized the Merkle hash tree techniques to obtain a better *k*-NN scheme that is secure against even a malicious cloud servers. There are also some studies that engage in location-based query processing over encrypted geospatial data (Lei et al., 2019; Lian et al., 2020). Lian et al. (2020) proposed an efficient *k*-NN scheme by employing the Moore curves together with the AES encryption scheme, that ensures the spatial data and location privacy.

The aforementioned studies use *k*-NN methods for either classification or query search applications. Unlike previous solutions, Kalideen, Osmanoglu & Tugrul (2019) proposed an efficient solution for the problem of computing the interpolation of *k*-NN to a given point in cloud computing settings. However, their solution reveals the knowledge of which data records were used to produce the interpolation to the cloud servers, and leaking such information might not be desired in some application required the data security. Unlike the protocol presented in Kalideen, Osmanoglu & Tugrul (2019), our protocol assures the desired security features, *i.e*. it hides data access pattern.

## Problem formulation

In this section, we will give more precise definition of the problem and its security requirements.

### Secure two-party *k*-NN interpolation problem

In our system there are two data owners DO_1_ and DO_2_ holding two different spatial databases D_1_ and D_2_, respectively. Each database D_*u*_ consists of *n* records $d_1^{(u)}, \ldots ,d_n^{(u)}$ such that each record $d_i^{(u)}$ is an *m*-dimensional spatial vector, *i.e*. $d_i^{(u)} = \left\langle d_{i,1}^{(u)}, \ldots ,d_{i,m}^{(u)}\right\rangle$ where *u* = 1,2. There are also two cloud pairs (CSP_1_^(*u*)^, CSP_2_^(*u*)^) so that each one is associated with a public key-secret key pair (*pk*_*u*_, *sk*_*u*_) of a public key encryption scheme that is semantically secure (Goldwasser & Micali, 1982). As the most of the studies in this field, we also consider each pair of cloud service providers (CSP_1_^(*u*)^, CSP_2_^(*u*)^) as two non-colluding cloud servers, *i.e*. CSP_1_^(*u*)^ stores the database and performs most of the homomorphic operations; on the other hand, CSP_2_^(*u*)^ keeps the secret key and helps CSP_1_^(*u*)^ to perform the complex operations over the ciphertexts.

In our problem, we assume that each data owner DO_*u*_ initially encrypts his database D_*u*_ as $E_{pk_u}(D_u)$ where $E_{pk_u}(D_u)$ consists of the attribute-wise encryptions ${E_{p{k_u}}}\left(d_{i,j}^{(u)}\right)$ for 1 ≤ *i* ≤ *n* and 1 ≤ *j* ≤ *m*. Each DO_*u*_ then outsources $E_{pk_u}(D_u)$ together with the query processing service to CSP_1_^(*u*)^. Note that the underlying public key encryption scheme should enable cloud servers to perform homomorphic operations over ciphertexts.

There is also an authorized query owner QO who wants to retrieve the interpolation of *k*-nearest neighbors of a query point Q from both databases D_1_ and D_2_ stored in CSP_1_^(1)^ and CSP_1_^(2)^, respectively. After QO requests the interpolation, the cloud service providers generate the result by performing required operations over the encrypted databases. This process should output the interpolation of *k*-nearest neighbors only to the query owner. The query owner should not learn any information other than the interpolation during this process. We denote such process as secure two-party *k*-nearest neighbors (STPkNN) protocol. We remark that STPkNN protocol should preserve the confidentiality of the records in the databases D_1_ and D_2_, and protect the privacy of the query point. Moreover, the protocol should hide data access patterns, *i.e*. it should not reveal the information of which data records were used to produce the interpolation of *k*-nearest neighbors to any data owner or any cloud service provider.

### Example

In 2016, European Union adopted a new regulation on the protection of personal data, Regulation (EU) 2016/679 of the European Parliament and Of The Council (European-Parliament, 2016). The regulation states that ‘the protection of natural persons in relation to the processing of personal data is a fundamental right’. All of the personal health records that reveal information relating to the past, current or future physical or mental health status of the data subject are considered as personal sensitive data in the regulation. Therefore, the personal health records should be protected against unauthorized parties, *i.e*. only the one approved by the owner should be able to access to the data.

On the other hand, the processing of health data may be significant to advance research or healthcare practices. Consider a doctor who tries to determine whether a person has a particular hearth disease or not by analyzing the medical records of the person. In addition, the doctor may desire to compare the patient’s medical records with other patients’ presenting similar properties in order to improve diagnostic accuracy. In fact, this comparison enables the doctor to evaluate the validity of some tests, especially when the scores do not match the expected values. Consequently, the doctor can make an accurate diagnosis, if he is allowed to reach the data of other patients in the same region or across the country. Moreover, if the personal health records are stored in the cloud as encrypted in order not to violate the fundamental right of the owner of the records, it will be possible to perform reliable analysis on large datasets.

Let us clarify it with an example. Consider the subset of heart disease data set from UCI Machine Learning Repository depicted in Table 1. There are 10 different instances shown in the table, and each instance is associated with five attributes: ID (patient’s identity), trestbps (resting blood pressure in mm Hg), chol (serum cholesterol in mg/dl), thalach (maximum heart rate achieved), and oldpeak (ST depression induced by exercise relative to rest). Assume the data owner, which can be viewed as hospital in this context, encrypts these attributes, and outsources the encrypted database *E*_*pk*_(*D*) together with the future query processing to the cloud. Also, assume there is a doctor who wants to determine whether a specific patient carries risk for a particular hearth disease. Let the medical record of the patient be ${Q} = \langle 150, 250, 145, 3\rangle$. The doctor, that will be the query owner in our context, asks the interpolation of *k*-nearest neighbors of Q from the cloud by providing the encryption *E*_*pk*_(*Q*) to the cloud. Then, the cloud determines the interpolation of *k*-nearest neighbors by searching the encrypted database *E*_*pk*_(*D*). For simplicity, let *k* be 3 for this example. As we observe here, the instances having IDs 1, 7, and 9 will be the 3 nearest neighbors to Q. So, the cloud returns the interpolation $T = \langle 141.6, 251.6, 152.3, 2.4 \rangle$ to the doctor that will benefit from T to make an accurate diagnosis. Consequently, necessary analysis can be carried out without revealing any sensitive information about both his patient and the other patients.

**Table 1:** A subset heart disease data set.

ID	trestbps	chol	thalach	oldpeak
1	145	233	150	2.3
2	160	286	108	1.5
3	120	229	129	2.6
4	130	250	187	3.5
5	130	204	172	1.4
6	120	236	178	0.8
7	140	268	160	3.6
8	120	354	163	0.6
9	130	254	147	1.4
10	140	203	155	3.1

### Effect of collaboration on interpolation accuracy

Aguilar et al. (2005) stated that if interpolation models are developed with an insufficient amount of data, they will be less accurate and reliable. Namely, the collaboration between participants affects the accuracy of interpolation models. We here conduct a series of experiments to assess the impact of collaboration between participants on the accuracy of prediction in the interpolation methods. In our experiments, we employ two publicly available datasets from U.S. National Geochemical Survey Database that present sodium (Na) content of the soil in two states: Colorado and Wisconsin. Summary statistics of both data sets are presented in Table 2.

**Table 2:** Summary statistics of data sets.

	Colorado	Wisconsin
Mean	0.999	0.773
Median	0.912	0.771
Minimum	0.063	0.007
Maximum	3.230	2.043
Standard Deviation	0.460	0.299
Skewness	0.912	−0.177
Kurtosis	4.034	3.131

**Figure 1:**
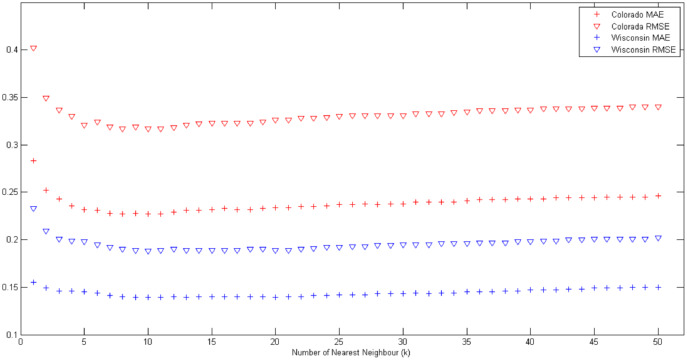
Effects of varying *k* values on MAE and RMSE for Colorado and Wisconsin data sets.

There are various performance evaluation metrics for interpolation methods. We here employed Mean Absolute Error (MAE) and Root Mean Squared Error (RMSE), which are often chosen as evaluation metrics for numerical prediction. The small values of both MAE and RMSE indicate that models will produce results that are more accurate. MAE and RMSE values are calculated as follows;

(1) $${MAE} = \left[ {\displaystyle{1 \over n}\sum\limits_{i = 1}^n |p({x_i},{y_i}) - a({x_i},{y_i})|} \right]$$

(2) $${ RMSE} = {\left[ {\displaystyle{1 \over n}\sum\limits_{i = 1}^n {{\left( {p({x_i},{y_i}) - a({x_i},{y_i})} \right)}^2}} \right]^{1/2}}$$

where *n* is the total number of data points in the dataset, *p*({*x*_*i*_, *y*_*i*_}) and *z*({*x*_*i*_, *y*_*i*_}) are predicted and actual values at location ({*x*_*i*_, *y*_*i*_}), respectively. The effects of varying *k* values on MAE and RMSE values are shown in Fig. 1.

We assume that two data holders share all data points in the data set. Both data sets are randomly divided into two parts using sampling without replacement strategy, assuming each party has one of the pieces. In some situations, data holders may not have data in equal proportions. So, we have determined different sharing ratios considering the cases where there is no equal distribution. We specify the *β* value as the distribution ratio, which means that if one party holds *β* portion of the data, the other party will hold the remaining portion (1 − *β*). After several trials, the MAE and RMSE values obtained according to the various number of nearest neighbor counts are shown in the Tables 3 and 4, respectively.

**Table 3:** Effects of collaboration for varying *k* and *β* (split ratio between parties) values on MAE.

*β*	Wisconsin				Colorado			
*k*	25	50	75	100	25	50	75	100
1	0.183	0.169	0.163	0.155	0.319	0.298	0.290	0.283
5	0.156	0.147	0.146	0.145	0.258	0.241	0.235	0.232
10	0.158	0.145	0.143	0.139	0.258	0.242	0.234	0.227
15	0.161	0.147	0.142	0.140	0.259	0.245	0.236	0.232
30	0.167	0.154	0.149	0.143	0.269	0.252	0.244	0.238
50	0.181	0.161	0.154	0.150	0.286	0.262	0.252	0.246

**Table 4:** Effects of collaboration for varying *k* and *β* (split ratio between parties) values on RMSE.

*β*	Wisconsin				Colorado			
*k*	25	50	75	100	25	50	75	100
1	0.254	0.243	0.241	0.233	0.443	0.421	0.408	0.402
5	0.208	0.199	0.199	0.198	0.361	0.334	0.327	0.321
10	0.212	0.196	0.193	0.188	0.358	0.337	0.325	0.317
15	0.215	0.198	0.193	0.189	0.358	0.341	0.329	0.323
30	0.224	0.206	0.200	0.195	0.368	0.348	0.338	0.331
50	0.238	0.214	0.207	0.202	0.385	0.360	0.347	0.340

As seen from the results, the smallest MAE and RMSE values are observed when the 10-nearest neighbors are used for all points in each data set. The smallest MAE and RMSE are underlined in the tables. As seen from the Table 3, if only half of the data is available for creating a prediction model, there will be a deterioration in MAE values of 4.31% for the Wisconsin data set and 6.60% for the Colorado data set. It is also possible to observe similar aspects in Table 4 for each split ratio. As observed from the results, the data holder who has less amount of data always produces less accurate predictions. On the contrary, if there is a sufficient amount of data, the predictions generated by the model are more accurate and reliable.

## Premilinaries

In this section, we will present the notations and the definitions of some primitives that will be used in our proposed protocols.

### Notation

We here give the notations used in this paper.

- *n*, the number of records in each database
- *m*, the number of the attributes in each record
-
$\ell$, the domain size (in bits) of the squared Euclidean distance
- DO_*u*_, the *u*^*th*^ data owner
- D_*u*_, the *u*^*th*^ database
-
$d_i^{(u)}$, the *i*^*th*^ record of the database D_*u*_
- QO, the query owner
- Q, the query point
- [*x*], the encryption of the individual bits of *x*
-
$d_{min_p}$, the *p*^*th*^ closest record to Q
- (*pk*_*u*_, *sk*_*u*_), the public key-secret key pair assigned to the *u*^*th*^ cloud pair
- (CSP_1_^(*u*)^, CSP_2_^(*u*)^), the *u*^*th*^ cloud pair, *i.e*. the former holds the encryption of the database $E_{pk_u}(D_u)$ and the latter holds the corresponding secret key *sk*_*u*_.

### Homomorphic encryption

Homomorphic encryption is an encryption scheme that allows users to perform some mathematical operations on ciphertexts, such as addition and multiplication. This property enables to protect the confidentiality of the data, and makes the encryption scheme a very practical and useful tool in cloud computing, especially for the sensitive data. For that reason, homomorphic encryption schemes have been gaining a lot of attention in recent years. Within this direction, many homomorphic encryption schemes have been proposed (Goldwasser & Micali, 1982; Elgamal, 1984; Boneh, Goh & Nissim, 2005). In this study we use a well-known homomorphic encryption system, the Paillier scheme, to construct our protocols.

Let *E*_*pk*_(·) be the encryption function with the public key *pk* and *D*_*sk*_(·) be the decryption function with the secret key *sk*. For any given two plaintexts *a* and *b*, the Paillier scheme satisfies the following properties:

- Addition: *D*_*sk*_(*E*_*pk*_(*a* + *b*)) = *D*_*sk*_(*E*_*pk*_(*a*) * *E*_*pk*_(*b*) *mod N*^2^)
- Multiplication: *D*_*sk*_(*E*_*pk*_(*a* * *b*)) = *D*_*sk*_(*E*_*pk*_(*a*)^*b*^*mod N*^2^)

Note that the Paillier encryption scheme is semantically secure (Paillier, 1999).

### Basic security primitives

Here, we briefly explain a set of basic security protocols. In these protocols, it’s assumed that there exist two semi-honest parties *P*_1_ and *P*_2_ joining the protocols, and the Paillier’s secret key is known only to one of them. We will also introduce two new security primitives in “Construction” that will be employed together with the basic primitives given here as building blocks in forming our construction.

#### Secure multiplication (SM) protocol

Consider two parties P_1_ and P_2_ such that the former holds (*E*_*pk*_(*x*), *E*_*pk*_(*y*)) and the latter holds the secret key *sk*, where *x* and *y* are not known to both parties. The protocol outputs *E*_*pk*_(*x* * *y*) to P_1_. Note that the output *E*_*pk*_(*x* * *y*) is only known to P_1_, and no information about *x* and *y* is revealed to any party during the protocol.

#### Secure squared Euclidean distance (SSED) protocol

The protocol considers two parties P_1_ and P_2_ with the inputs (*E*_*pk*_(*X*), *E*_*pk*_(*Y*)) and the secret key *sk*, respectively, and outputs *E*_*pk*_(|*X* − *Y*|^2^) to P_1_, where *X* and *Y* are *m* dimensional vectors. In the protocol, the encryption of squared Euclidean distance *E*_*pk*_(|*X* − *Y*|^2^) is only known to P_1_.

#### Secure bit-decomposition (SBD) protocol

The protocol considers P_1_ with the input *E*_*pk*_(*x*) and P_2_ with the secret key *sk*, and outputs the encryptions of the bit-decomposition of *x* as $[x] = \langle E_{pk} (x_1),\ldots ,E_{pk} (x_\ell)\rangle$, where $0 \le x \le 2^\ell$. Note that the encryptions of bit-decomposition [*x*] is known to only P_1_.

#### Secure minimum (SMIN) protocol

In the protocol, P_1_ with the inputs ([*x*], [*y*]) and P_2_ with the secret key *sk* securely compute the encryption of individual bits of minimum between *x* and *y* as [*min*(*x*,*y*)]. Note that the output [*min*(*x*,*y*)] is only known to P_1_, and no information about *x* and *y* is revealed to any party during the protocol.

#### Secure minimum out of *n* numbers (SMIN_n_) protocol

In the protocol, P_1_ with the inputs ([*x*_1_],…,[*x*_*n*_]) and P_2_ with the secret key *sk* securely compute [*min*(*x*_1_,…,*x*_*n*_)], where [*min*(*x*_1_,…,*x*_*n*_)] is the encryption of the individual bits of *min*(*x*_1_,…,*x*_*n*_). Note that the output [*min*(*x*_1_,…,*x*_*n*_)] is only known to P_1_, and no information about *x*_*i*_ for any *i* is revealed to any party during the protocol.

#### Secure Bit-OR (SBOR) protocol

Consider two parties P_1_ and P_2_ such that the former holds (*E*_*pk*_(*a*), *E*_*pk*_(*b*)) and the latter holds the secret key *sk*, where *a* and *b* are two bits. The protocol outputs $E_{pk}(a \vee  b)$ to *P*_1_. The output $E_{pk} (a\vee b)$ is only known to P_1_, and no information about a and b is revealed to any party during the protocol.

Since we don’t aim to study the existing protocols given above, we simply consider the most efficient implementation of them which were presented in Elmehdwi, Samanthula & Jiang (2014) and Samanthula, Hu & Jiang (2013). However, the implementation of the SMIN_*n*_ protocol given in Elmehdwi, Samanthula & Jiang (2014) fails for some inputs, *i.e*. it generates an incorrect output if the size of the input is given as *n* = 8*k* + 1 for some *k* ∈ *Z*. Let me illuminate it with an example: assume the protocol takes nine inputs ([*x*_1_],…,[*x*_9_]). At the last step, the protocol applies the SMIN protocol to the intermediate values [*x*′_1_] and [*x*′_7_]) (the encryptions of the local minimums), and outputs the encryption of 0 since [*x*′_7_] was set to the encryption of zero at some previous steps. Therefore, independent of the inputs, the protocol always outputs the encryption of zero as the final output when the size of the input is given as *n* = 8*k* + 1 for some *k* ∈ *Z*. Thus, we develop a new implementation of the SMIN_*n*_ protocol that simply works as follows:

1. The server P_1_ initially executes the SMIN protocol together with P_2_ on [*x*_1_] and [*x*_2_] to get [*R*_1_] = [*min*(*x*_1_,*x*_2_)] as the encryption of the individual bits of *min*(*x*_1_,*x*_2_),
2. it then iteratively runs the SMIN protocol together with P_2_ on [*R*_*i*−1_] and [*x*_*i*+1_] to get [*R*_*i*_] = [*min*(*R*_*i*−1_,*x*_*i*+1_)] as the encryption of the individual bits of *min*(*x*_1_,*x*_2_,…,*x*_*i*+1_) for *i* = 2… (*n* − 1).

Note that the final output of the iterative steps will be [*R*_*n*−1_] = [*min*(*x*_1_,…,*x*_*n*_)], which is the encryption of the individual bits of *min*(*x*_1_,…,*x*_*n*_).

## Construction

In this section, we first introduce two new security primitives: the Secure bit-AND-OR (SBAOR) protocol and Secure Transformation (ST) protocol. We then give the security analysis of this protocols. By utilizing SBAOR and ST protocols together with the basic security primitives given in “Premilinaries”, we construct our main protocol. Furthermore, we also give the security analysis of the main protocol and discuss the computation complexity at the end of this section.

### Secure bit-AND-OR (SBAOR) protocol

The SBAOR protocol allows the servers to securely compute the negation of the logical disjunction of all bitwise multiplications *x*_*i*_ · *y*_*i*_ in encrypted form without revealing the bit vectors to any party. In the main protocol, it will help the servers to separate the index of the current closest record to the query point from all other records of both databases by assigning the encryption of 1 to that particular index and the encryption of 0 to all other indices. In this way, the servers will be able to calculate the current closest record, and remove the corresponding index from further calculations.

The protocol considers two parties P_1_ and P_2_ such that the former holds ([*x*], [*y*]) and the latter holds the secret key *sk*, where [*x*] and [*y*] are the encryption of individual bits of *x* and *y*. The protocol enables the parties P_1_ and P_2_ to securely compute the encryption ${E_{pk}}(\overline A )$ where $\overline A = 1 - A$ and $A = (x_1 \cdot y_1)\vee (x_2 \cdot y_2)\vee \cdots \vee (x_\ell \cdot y_\ell)$. The output ${E_{pk}}(\overline A )$ is only known to P_1_, and no information about *x* and *y* is revealed to any party during the protocol.

In the protocol, P_1_ and P_2_ first runs the SM protocol on the inputs *E*_*pk*_(*x*_*i*_) and *E*_*pk*_(*y*_*i*_) to calculate *E*_*pk*_(*x*_*i*_ * *y*_*i*_) for $i\in [\ell]$ where *x*_*i*_ and *y*_*i*_ are the i-th bits of *x* and *y*, respectively. Note that each *E*_*pk*_(*x*_*i*_ * *y*_*i*_) is only revealed to P_1_. The server P_1_ then calculates $E_{pk} (x_1 \cdot y_1 \lor \cdots \lor x_{\ell} \cdot y_{\ell})$ as follows:

- it initially executes the SBOR protocol together with P_2_ on *E*_*pk*_(*x*_1_ * *y*_1_) and *E*_*pk*_(*x*_2_ * *y*_2_) to get $E_{pk}(R_1) = E_{pk}(x_1*y_1 \lor x_2*y_2)$,
- it then iteratively runs the SBOR protocol together with P_2_ on *E*_*pk*_(*R*_*i*−1_) and *E*_*pk*_(*x*_*i*+1_ * *y*_*i*+1_) to get $E_{pk}(R_i) = E_{pk}(R_{i-1} \lor x_{i+1}*y_{i+1})\ {\rm for}\ i=2 \cdots \ell-1$.

Note that the final output of the iterative steps will be $E_{pk}(R_{\ell-1}) = E_{pk} (x_1 \cdot y_1 \lor \cdots \lor x_{\ell} \cdot y_{\ell})$. Finally, P_1_ applies the equation ${E_{pk}}(\overline {{R_{\ell - 1}}} ) = {E_{pk}}(1)*{E_{pk}}{({R_{\ell - 1}})^{N - 1}}$ to compute the final output.

**Algorithm 1:** SBAOR.

**Input:** ([*x*], [*y*]) from P_1_ and *sk* from P_2_
**Output:** ${E_{pk}}(\overline {x \cdot y} )$ to P_1_
1. P_1_ and P_2_;
**for***i* = 1 to $\ell$**do**
${E_{pk}}({x_i}*{y_i}) \leftarrow {\rm SM}({E_{pk}}({x_i}),{E_{pk}}({y_i}))$;
2. P_1_ and P_2_;
${E_{pk}}({R_1}) \leftarrow {\rm SBOR}({E_{pk}}({x_1}*{y_1}),{E_{pk}}({x_2}*{y_2})$;
**for***i* = 2 to $\ell$ − 1 **do**
${E_{pk}}({R_i}) \leftarrow {\rm SBOR}({E_{pk}}({R_{i - 1}}),{E_{pk}}({x_{i + 1}}*{y_{i + 1}}))$;
3. P_1_;
${E_{pk}}(\overline {x \cdot y} ) \leftarrow {E_{pk}}(1 - {R_{\ell - 1}}) \leftarrow {E_{pk}}(1) \times {E_{pk}}{({R_{\ell - 1}})^{N - 1}}$;

*Security Analysis of SBAOR:* At the beginning of the protocol, the servers P_1_ and P_2_ execute the Secure Multiplication protocol. As emphasized in “Premilinaries”, the output of the protocol is only revealed to the server P_1_, and no information about the plaintexts *x*_*i*_ and *y*_*i*_ is revealed to any party during this protocol. Later, the servers run the Secure Bit-OR (SBOR) Protocol on the inputs *E*_*pk*_(*R*_*i*_) and *E*_*pk*_(*x*_*i*+1_ * *y*_*i*+1_). The SBOR protocol outputs the new *E*_*pk*_(*R*_*i*+1_) only to the server P_1_, and no information about the plaintexts is revealed to any party during the protocol. At the final, the server P_1_ only applies some homomorphic operations on the encryption $E_{pk}(R_{\ell-1})$ computed at the previous step. Therefore, the SBAOR protocol protects the confidentiality of the data, *i.e*. no information about the contents of the encryptions is revealed to any party during the protocol.

### Secure Transformation (ST) protocol

The ST Protocol enables the servers to transform the encryption of a record under a public key to the encryption of same record under another public key. In the main protocol, the servers employ ST Protocol to collect the encryptions of local minimums, that indicate the indexes of local closest records of both databases, under the same public key so that they can decide the minimum among them.

The protocol considers three parties ${\rm P}_1^{({u_1})}$ with the input $E_{pk_{u_1}}(t)$, ${\rm P}_2^{({u_1})}$ with the secret key $sk_{u_1}$, and ${\rm P}_1^{({u_2})}$. The protocol simply aims to transform the encryption of a record *t* under the public key $pk_{u_1}$ to the encryption of *t* under the public key $pk_{u_2}$. Note that no information about *t* is revealed to any party during the protocol and the output $E_{pk_{u_2}}(t)$ is only known to the party ${\rm P}_1^{({u_2})}$.

Briefly, ${\rm P}_1^{({u_1})}$ first masks $E_{pk_{u_1}}(t)$ with the randomly chosen vector ${r}^{\prime} \in Z_N^m$ as $\mu = E_{pk_{u_1}}(t) * E_{pk_{u_1}}(r^{\prime})$, and sends *μ* to ${\rm P}_2^{({u_1})}$ and $E_{pk_{u_2}}(r^{\prime})$ to ${\rm P}_1^{({u_2})}$. After getting *μ*, ${\rm P}_2^{({u_1})}$ first decrypts it as $\mu^{\prime} = D_{sk_{u_1}}(\mu)$, then encrypts *μ*′ with the public key $pk_{u_2}$ as $E_{pk_{u_2}}(\mu^{\prime})$, and finally sends the encryption to ${\rm P}_1^{({u_2})}$. After receiving the encryption, the party ${\rm P}_1^{({u_2})}$ first removes the randomness *r*′ from the encryption $E_{pk_{u_2}}(\mu^{\prime})$ and gets the encryption $E_{pk_{u_2}}(t)$ as $E_{pk_{u_2}}(t) = E_{pk_{u_2}}(\mu^{\prime} - r^{\prime})$. From the homomorphic property of the underlying encryption scheme, $E_{pk_{u_2}} (\mu^{\prime} - r^{\prime})$ can easily be calculated as $E_{pk_{u_2}}(\mu^{\prime})*E_{pk_{u_2}}(r^{\prime})^{N - 1}$.

**Algorithm 2:** ST.

**Input**: $E_{pk_{u_1}}(t)$ from P $_1^{({u_1})}$ and $sk_{u_1}$ from P $_2^{({u_1})}$
**Output:** $E_{pk_{u_2}}(t)$ to P $_1^{({u_2})}$
1. P $_1^{({u_1})}$;
$\mu \leftarrow {E_{p{k_{{u_1}}}}}(t) \times {E_{p{k_{{u_1}}}}}({r}^{\prime})$; ${r}^{\prime}{\ \in _R}\ Z_N^m$;
send *μ* to P $_2^{({u_1})}$ and $E_{pk_{u_2}}(r^{\prime})$ to P $_1^{({u_2})}$;
2. P $_2^{({u_1})}$;
${\mu }^{\prime} \leftarrow {D_{s{k_{{u_1}}}}}(\mu )$;
send $E_{pk_{u_2}}(\mu^{\prime})$ to P $_1^{({u_2})}$;
3. P $_1^{({u_2})}$;
${E_{p{k_{{u_2}}}}}(t) \leftarrow {E_{p{k_{{u_2}}}}}({\mu }^{\prime} - {r}^{\prime}) \leftarrow {E_{p{k_{{u_2}}}}}({\mu }^{\prime}) \times {E_{p{k_{{u_2}}}}}{({r}^{\prime})^{N - 1}}$;

*Security Analysis of ST:* At the beginning of the protocol, the servers ${\rm P}_1^{({u_1})}$ randomizes the encryption $E_{pk_{u_1}}(t)$ with ${r}^{\prime} \in Z_N^m$ before sending it to the server ${\rm P}_2^{({u_1})}$. So, the decryption computed by ${\rm P}_2^{({u_1})}$ will be uniformly random in $Z_N^m$. Besides, P $_1^{({u_2})}$ locally subtracts the encryption of the randomness *r*′ under the public key $pk_{u_2}$ from the encryption sent by ${\rm P}_2^{({u_1})}$ by performing some homomorphic operations. Thus, the protocol does not reveal any information about the record *t* to any party.

### Main protocol

**Figure 2:**
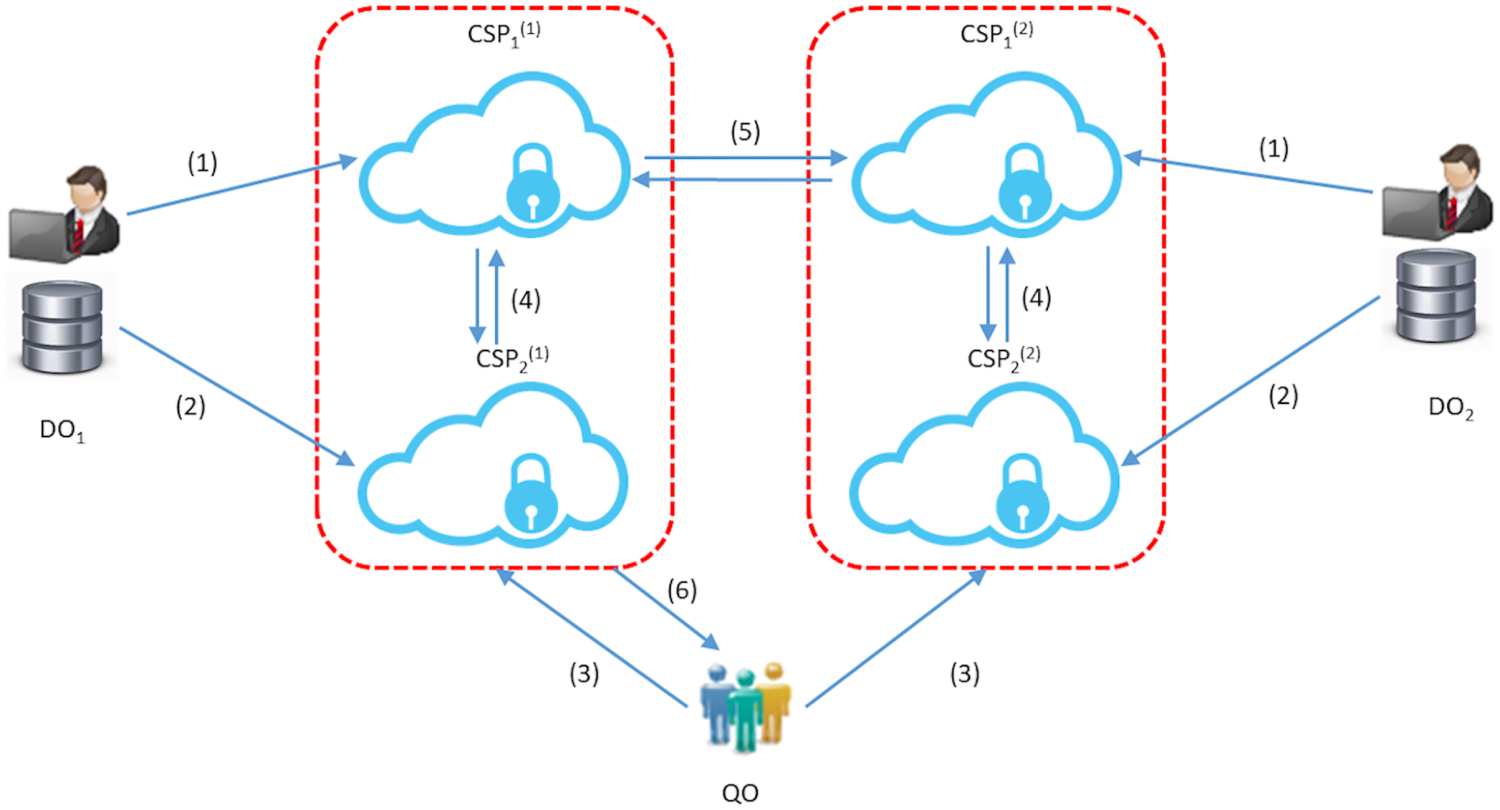
The STPkNN protocol: for *u* = 1, 2; (1) each DO_*u*_ uploads its data to the server CSP_1_^(*u*)^; (2) each DO_*u*_ gives its secret key to CSP_2_^(*u*)^; (3) QO sends its query point Q in encrypted form to the servers CSP_1_^(*u*)^; (4) CSP_1_^(*u*)^ and CSP_2_^(*u*)^ find the local nearest neighbours; (5) CSP_1_^(1)^ and CSP_1_^(2)^ decide on the global nearest neighbour among the local nearest neighbors (4 and 5 are repeated *k* times in the protocol); (6) the final prediction value is forwarded to QO.

In this section, we will give the construction of our main protocol that enables a query owner to extract the interpolation of *k*-nearest neighbors for a query point of his choice as shown in Fig. 2. As we stated in the “Introduction”, our construction can be viewed as an extension of the protocol presented in Elmehdwi, Samanthula & Jiang (2014) that proposes an efficient solution of the *k*-nearest neighbor query problem over encrypted database outsourced to a single cloud.

We assume that each data owner DO_*u*_ has a database D_*u*_ that consists of *n* records $d_1^{(u)}, \ldots ,d_n^{(u)}$ where each $d_i^{(u)}$ is *m*-dimensional vector that lies in $[0,2^\ell]$. We also assume that there exist two non-colluding semi-honest cloud service providers, ${\rm CSP}_1^{(u)}$ and ${\rm CSP}_2^{(u)}$ for each database D_*u*_ where ${\rm CSP}_1^{(u)}$ is given the encryption of the database D_*u*_ and ${\rm CSP}_2^{(u)}$ is given the corresponding secret key *sk*_*u*_.

Initially, each DO_*u*_ encrypts his database D_*u*_ as ${E_{p{k_u}}}\left(d_{i,j}^{(u)}\right)$ where 1≤ *i* ≤ *n* and 1≤ *j* ≤ *m*. Each DO_*u*_ then outsources the encryptions of the database, together with the future query service to the clouds, *i.e*. DO_*u*_ gives ${E_{p{k_u}}}\left(d_{i,j}^{(u)}\right)$ to ${\rm CSP}_1^{(u)}$ and his secret key *sk*_*u*_ to ${\rm CSP}_2^{(u)}$. When the query owner (QO) wants to retrieve the interpolation of the *k*-nearest neighbors for a query point Q, he produces two encryptions of his query point Q as $E_{pk_1}({Q})= \langle E_{pk_1} (q_1),\ldots,E_{pk_1} (q_m) \rangle$ and $E_{pk_2}({Q})= \langle E_{pk_2} (q_1),\ldots,E_{pk_2} (q_m) \rangle$ using the public keys of the data owners DO_1_ and DO_2_, respectively; and gives each encryption $E_{pk_u} ({Q})$ to the corresponding cloud service provider ${\rm CSP}_1^{(u)}$.

After receiving the encryption $E_{pk_u}({Q})$, each ${\rm CSP}_1^{(u)}$ runs the SSED protocol together with the corresponding server ${\rm CSP}_2^{(u)}$ on the input $\left({E_{p{k_u}}}({Q}),{E_{p{k_u}}}\left(d_i^{(u)}\right)\right)$ where $E_{pk_u}\left(d_i^{(u)}\right)= \left\langle E_{pk_u} \left(d_{i,1}^{(u)} \right),\ldots,E_{pk_u} \left(d_{i,m}^{(u)}\right) \right\rangle$ for 1 ≤ *i* ≤ *n*, and obtains the encryption of the squared Euclidean distance between Q and $d_i^{(u)}$ as ${E_{p{k_u}}}\left(e_i^{(u)}\right)$ where $e_i^{(u)} = |{Q} - d_i^{(u)}{|^2}$. From the SSED protocol, ${E_{p{k_u}}}\left(e_i^{(u)}\right)$ is revealed only to ${\rm CSP}_1^{(u)}$.

Opposite to the protocol proposed in Kalideen, Osmanoglu & Tugrul (2019), instead of sending the encryptions ${E_{p{k_u}}}\left(e_i^{(u)}\right)$ to the server ${\rm CSP}_2^{(u)}$ where 1 ≤ *i* ≤ *n*, that reveals the information of which indexes being used to compute the interpolation to ${\rm CSP}_2^{(u)}$, each ${\rm CSP}_1^{(u)}$ securely runs the SBD protocol with the server ${\rm CSP}_2^{(u)}$ on the inputs ${E_{p{k_u}}}\left(e_i^{(u)}\right)$ to compute $[{e_i}(u)] = \left\langle {E_{p{k_u}}}\left(e_{i,1}^{(u)}\right), \ldots ,{E_{p{k_u}}}\left(e_{i,\ell }^{(u)}\right)\right\rangle$, the encryptions of the individual bits of $e_i^{(u)}$. Note that $\left[e_i^{(u)}\right]$ is only revealed to ${\rm CSP}_1^{(u)}$.

After this stage, the servers produce the interpolation of the *k*-nearest neighbours of the query point Q in an iterative way. In each iteration:

- Each pair of servers ${\rm CSP}_1^{(u)}$ and ${\rm CSP}_2^{(u)}$ securely calculate the encryptions of the individual bits of the minimum value $\left[e_{min}^{(u)}\right]$ among $\left[e_1^{(u)}\right], \ldots ,\left[e_n^{(u)}\right]$ by running the protocol SMIN_*n*_. Note that $\left[e_{min}^{(u)}\right]$ is only revealed to ${\rm CSP}_1^{(u)}$.
- Each ${\rm CSP}_1^{(u)}$ then locally calculates the encryption of *e*_*min*_ from $\left[e_{min}^{(u)}\right]$ as

${E_{p{k_u}}}\left(e_{min}^{(u)}\right) = \prod\limits_{i = 0}^{\ell - 1} {E_{p{k_u}}}{\left(e_{min,i}^{(u)}\right)^{{2^{\ell - i - 1}}}} = {E_{p{k_u}}}\left(e_{min,1}^{(u)} \times {2^{\ell - 1}} + \ldots + e_{min,\ell }^{(u)}\right).$

- At this stage of the protocol, the servers ${\rm CSP}_1^{(1)}$ and ${\rm CSP}_1^{(2)}$ have ${E_{p{k_1}}}\left(e_{min}^{(1)}\right)$ and ${E_{p{k_2}}}\left(e_{min}^{(2)}\right)$ as the encryption of the minimum distances. Now, the servers apply the following steps to decide the minimum among $e_{min}^{(1)}$ and $e_{min}^{(2)}$:

– the servers ${\rm CSP}_1^{(2)}$ with the input ${E_{p{k_2}}}\left(e_{min}^{(2)}\right)$, ${\rm CSP}_2^{(2)}$ with the secret key *sk*_2_, and ${\rm CSP}_1^{(1)}$ securely runs the ST protocol to compute the encryption of $e_{min}^{(2)}$ under the public key *pk*_1_. Note that ${E_{p{k_1}}}\left(e_{min}^{(2)}\right)$ is only known to ${\rm CSP}_1^{(1)}$.
– the server ${\rm CSP}_1^{(1)}$ now executes the SBD protocol with the server ${\rm CSP}_2^{(1)}$ on the inputs ${E_{p{k_1}}}\left(e_{min}^{(1)}\right)$ and ${E_{p{k_1}}}\left(e_{min}^{(2)}\right)$ to compute $\left[e_{min}^{(1)}\right]$ and $\left[e_{min}^{(2)}\right]$ where $\left[e_{min}^{(u)}\right] = \left\langle {E_{p{k_1}}}\left(e_{min,1}^{(u)}\right), \ldots ,{E_{p{k_1}}}\left(e_{min,\ell }^{(u)}\right)\right\rangle$. From the SBD protocol, $[e_{min}^{(1)}]$ and $[e_{min}^{(2)}]$ are only revealed to ${\rm CSP}_1^{(1)}$.
– ${\rm CSP}_1^{(1)}$ then runs the SMIN protocol with ${\rm CSP}_2^{(1)}$ on the inputs $[e_{min}^{(1)}]$ and $[e_{min}^{(2)}]$, and gets $[{e_{min}}] = \left[min\left\{ e_{min}^{(1)},e_{min}^{(2)}\right\} \right]$.
– after getting [*e*_*min*_], ${\rm CSP}_1^{(1)}$ locally calculates the encryption of *e*_*min*_ as

${\hskip2.2pc}{E_{p{k_1}}}({e_{min}}) = \prod\limits_{i = 0}^{\ell - 1} {E_{p{k_1}}}{({e_{min,i}})^{{2^{\ell - i - 1}}}}.$

– the servers ${\rm CSP}_1^{(1)}$ with the input $E_{pk_1}(e_{min})$, ${\rm CSP}_2^{(1)}$ with the secret key *sk*_1_, and ${\rm CSP}_1^{(2)}$ securely runs the ST protocol to compute the encryption of *e*_*min*_ under the public key *pk*_2_. Note that $E_{pk_2}(e_{min})$ is only known to ${\rm CSP}_1^{(2)}$.

- After identifying the minimum *e*_*min*_ among $e_{min}^{(1)}$ and $e_{min}^{(2)}$, each ${\rm CSP}_1^{(u)}$ locally computes the encryption of difference $\left({e_{min}} - e_i^{(u)}\right)$ for each *i* as ${E_{p{k_u}}}\left(\lambda _i^{(u)}\right) = {E_{p{k_u}}}\left({e_{min}} - e_i^{(u)}\right) = {E_{pk}}({e_{min}})*{E_{pk}}{\left(e_i^{(u)}\right)^{N - 1}}$.
- Each ${\rm CSP}_1^{(u)}$ then randomizes ${E_{p{k_u}}}\left(\lambda _i^{(u)}\right)$ as ${E_{p{k_u}}}\left(\alpha _i^{(u)}\right) = {E_{p{k_u}}}{\left(\lambda _i^{(u)}\right)^{r_i^{(u)}}} = {E_{p{k_u}}}\left(\lambda _i^{(u)}*r_i^{(u)}\right)$ where $r_i^{(u)}$ is a random number in *Z*_*N*_. It is a fact that only one is the encryption of zero among all 2*n* encryptions ${E_{p{k_u}}}(\alpha _i^{(u)})$ and all others are the encryptions of some random numbers where *i* = 1 … *n* and *u* = 1, 2.
- Each ${\rm CSP}_1^{(u)}$ securely runs the SBD protocol with the server ${\rm CSP}_2^{(u)}$ on the inputs ${E_{p{k_u}}}\left(\alpha _i^{(u)}\right)$ to compute $\left[\alpha _i^{(u)}\right] = \left\langle {E_{p{k_u}}}\left(\alpha _{i,1}^{(u)}\right), \ldots ,{E_{p{k_u}}}\left(\alpha _{i,\ell }^{(u)}\right)\right\rangle$, the encryptions of the individual bits of $\alpha _i^{(u)}$. Note that $\left[\alpha _i^{(u)}\right]$ is only revealed to ${\rm CSP}_1^{(u)}$.
- After getting the encryptions $\left[\alpha _i^{(u)}\right]$, each ${\rm CSP}_1^{(u)}$ runs the SBAOR protocol with the server ${\rm CSP}_2^{(u)}$ on $\left[\alpha _i^{(u)}\right]$ and [1] for each *i* where $[1] = \langle E_{pk_u}(1),\ldots,E_{pk_u}(1) \rangle$, and gets ${E_{p{k_u}}}\left(\beta _i^{(u)}\right) = {E_{p{k_u}}}\left(\overline {\alpha _i^{(u)} \cdot 1} \right)$. Note that one of the encryptions among all ${E_{p{k_u}}}\left(\beta _i^{(u)}\right)$ is $E_{pk_u}(1)$ and the remaining encryptions are $E_{pk_u}(0)$ where *i* ∈ [*n*] and *u* = 1,2. Furthermore, if $\beta _j^{(v)} = 1$, then $d_j^{(v)}$ is the closest record to Q from both databases.
-
${\rm CSP}_1^{(u)}$ securely runs the SM protocol with ${\rm CSP}_2^{(u)}$ on the inputs ${E_{p{k_u}}}\left(\beta _i^{(u)}\right)$ and ${E_{p{k_u}}}\left(d_{i,j}^{(u)}\right)$ to compute $\beta _{i,j}^{\prime (u)} = {E_{p{k_u}}}\left(\beta _i^{(u)}*d_{i,j}^{(u)}\right)$, for 1 ≤ *i* ≤ *n* and 1 ≤ *j* ≤ *m*. Then, each ${\rm CSP}_1^{(u)}$ can now calculate the encryption of its candidate for the first closest record $d_1^{\prime (u)}$ as ${E_{p{k_u}}}\left(d_1^{\prime (u)}\right) = \left\langle {E_{p{k_u}}}\left(d_{1,1}^{\prime (u)}\right), \ldots ,{E_{p{k_u}}}\left(d_{1,m}^{\prime (u)}\right)\right\rangle$ where ${E_{p{k_u}}}\left(d_{1,j}^{\prime (u)}\right) = \prod\nolimits_{i = 1}^n \beta _{i,j}^{\prime (u)}$. As we stated before, since only one of the encryptions among all ${E_{p{k_u}}}\left(\beta _i^{(u)}\right)$ is $E_{pk_u}(1)$ and the remaining are $E_{pk_u}(0)$, one of the encryptions ${E_{p{k_u}}}\left(d_1^{\prime (u)}\right)$ will be the encryption of zero and the other one will be the encryption of nonzero number that will be the first closest record.
-
${\rm CSP}_1^{(2)}$ with the input ${E_{p{k_2}}}(d_1^{\prime(2)})$, ${\rm CSP}_2^{(2)}$ with the secret key *sk*_2_, and ${\rm CSP}_1^{(1)}$ securely runs the ST protocol to compute the encryption of $d_1^{\prime (2)}$ under the public key *pk*_1_. Note that ${E_{p{k_1}}}\left(d_1^{\prime (2)}\right)$ is only known to ${\rm CSP}_1^{(1)}$.
-
${\rm CSP}_1^{(1)}$ now can calculate the encryption of the first closest record as ${E_{p{k_1}}}({d_{mi{n_1}}}) = {E_{p{k_1}}}\left(d_1^{\prime (1)}\right)*{E_{p{k_1}}}\left(d_1^{\prime (2)}\right)$. From the homomorphic property of the underlying encryption scheme, ${E_{p{k_1}}}\left(d_1^{\prime (1)}\right)*{E_{p{k_1}}}\left(d_1^{\prime (2)}\right) = {E_{p{k_1}}}\left(d_1^{\prime (1)} + d_1^{\prime (2)}\right)$, and since one of them is zero, ${E_{p{k_1}}}\left(d_1^{\prime (1)} + d_1^{\prime (2)}\right)$ will be the encryption of the first closest record from both databases.
- As the final step of the first iteration, the first closest records *d*_*min*1_ should be excluded from the further iterations. To this aim, each ${\rm CSP}_1^{(u)}$ securely executes the SBOR protocol with ${\rm CSP}_2^{(u)}$ on the inputs $\beta _i^{(u)}$ and ${E_{p{k_u}}}\left(e_{i,h}^{(u)}\right)$ where 1 ≤ *i* ≤ *n* and $1 \le h \le \ell$. As the output of the protocol, ${\rm CSP}_1^{(u)}$ gets the encryptions of renewed distances as ${E_{p{k_u}}}\left(e_{i,h}^{(u)}\right) = {E_{p{k_u}}}\left(\beta _i^{(u)} \vee e_{i,h}^{(u)}\right)$. Observe that if $\beta _j^{(u)} = {E_{p{k_u}}}(1)$ for a particular *j*, the corresponding distance $e_j^{(u)}$ will take the maximum value, *i.e*. $\left[e_j^{(u)}\right] = \langle {E_{p{k_u}}}(1), \ldots ,{E_{p{k_u}}}(1)\rangle$. On the other hand, if $\beta _i^{(u)} = {E_{p{k_u}}}(0)$, the SBOR protocol will have no effect on $e_i^{(u)}$.

Because our protocol outputs the interpolation of the *k*-nearest neighbors of the query point Q, the server ${\rm CSP}_1^{(1)}$ does not need to keep all the nearest records separately. Instead, it gradually builds the interpolation, *i.e*. after each iteration, ${\rm CSP}_1^{(1)}$ adds the current closest record $E_{pk_1}(d_{{min}_p})$ to the previous sum $E_{pk_1}(S_{p-1}) = E_{pk_1}(d_{min_1} + \ldots + d_{min_{p-1}})$ as $E_{pk_1}(S_{p-1})*E_{pk_1}(d_{min_p})$, and gets the current sum $E_{pk_1}(S_p) = E_{pk_1}(d_{min_1} + \ldots + d_{min_P})$.

After *k* iterations, ${\rm CSP}_1^{(1)}$ will have the sum $E_{pk_1}(S_k) = E_{pk_1}(d_{min_1} + \ldots + d_{min_k})$ as the encryption of the sum of the *k*-nearest neighbors of the query point Q. ${\rm CSP}_1^{(1)}$ then computes the randomization of the encryptions as $\gamma_j = E_{pk_1}(S_{k,j})*E_{pk_1}(r_j)$ where *r*_*j*_ are random numbers in *Z*_*N*_ and 1 ≤ *j* ≤ *m*. ${\rm CSP}_1^{(1)}$ then sends *γ*_*j*_ to ${\rm CSP}_2^{(1)}$ and *r*_*j*_ to the query owner. Upon receiving *γ*_*j*_, ${\rm CSP}_2^{(1)}$ decrypts them as $\gamma _j^{'} = {D_{s{k_1}}}({\gamma _j})$ and sends the decryptions to the query owner. The query owner QO then computes the sum of *k*-nearest record as $S_{k,j}^{'} = \gamma _j^{'} - {r_j}$ where 1 ≤ *j* ≤ *m*. As the final step, QO computes the interpolation of *k*-nearest neighbors of Q as $\langle S_{k,1}^{\prime}/k, \ldots ,S_{k,m}^{\prime}/k\rangle$.

### Security analysis

In this section, we will give the security analysis of the protocol shown in Algorithm 3. As we emphasized above, the data owners encrypt their data before outsourcing them to the cloud. Since they use the Paillier encryption scheme which is semantically secure, the data is not leaked to any cloud service provider. On the other hand, at the first step of Algorithm 3, the query point Q is encrypted before given to the corresponding cloud service providers. Similarly, since the underlying encryption scheme (the Paillier cryptosystem) is semantically secure, the query point Q is not revealed to any data owner or any cloud service provider.

**Algorithm 3:** STPkNN.

**Input:** $E_{pk_1}(D_u)$ from ${\rm CSP}_1^{(u)}$; *sk*_*u*_ from ${\rm CSP}_2^{(u)}$; Q from QO
**Output:***T*, the interpolation of *k*-nearest neighbors of Q
1. QO;
a) compute $E_{pk_u}({Q}) = \langle E_{pk_u} (q_1), \ldots,E_{pk_u} (q_m)\rangle ;$
b) send each $E_{pk_u}({Q})$ to the corresponding server ${\rm CSP}_1^{(u)}$;
2. ${\rm CSP}_1^{(u)}$ and ${\rm CSP}_2^{(u)}$;
** for***i* = 1 to *n***do**
${E_{p{k_u}}}\left(e_i^{(u)}\right) \leftarrow {\rm SSED}\left({E_{p{k_u}}}({Q}),{E_{p{k_u}}}\left(d_i^{(u)}\right)\right)$;
$\left[e_i^{(u)}\right] \leftarrow {\rm SBD}\left({E_{p{k_u}}}\left(e_i^{(u)}\right)\right)$
3. **for***p* = 1 to *k***do**
a) ${\rm CSP}_1^{(u)}$ and ${\rm CSP}_2^{(u)}$;
– $[e_{min}^{(u)}] \leftarrow {\rm SMIN}_n([e_1^{(u)}], \ldots ,[e_n^{(u)}])$;
– ${\rm CSP}_1^{(u)}$ computes ${E_{p{k_u}}}\left(e_{min}^{(u)}\right) \leftarrow \prod\nolimits_{i = 0}^{\ell - 1} {E_{p{k_u}}}{\left(e_{min,i}^{(u)}\right)^{{2^{\ell - i - 1}}}}$;
b) ${\rm CSP}_1^{(1)}$, ${\rm CSP}_2^{(1)}$, ${\rm CSP}_1^{(2)}$, and ${\rm CSP}_2^{(2)}$;
– ${\rm CSP}_1^{(2)}$, ${\rm CSP}_2^{(2)}$, and ${\rm CSP}_1^{(1)}$ execute ${E_{p{k_1}}}\left(e_{min}^{(2)}\right) \leftarrow {\rm ST}\left({E_{p{k_2}}}\left(e_{min}^{(2)}\right)\right)$;
– ${\rm CSP}_1^{(1)}$ and ${\rm CSP}_2^{(1)}$ compute $\left[e_{min}^{(u)}\right] \leftarrow {\rm SBD}\left({E_{p{k_1}}}\left(e_{min}^{(u)}\right)\right)$;
– ${\rm CSP}_1^{(1)}$ and ${\rm CSP}_2^{(1)}$ compute $\left[{e_{min}}\right] \leftarrow {\rm SMIN}\left({E_{p{k_1}}}\left(\left[e_{min}^{(1)}\right],\left[e_{min}^{(2)}\right]\right)\right)$;
– ${\rm CSP}_1^{(1)}$ computes ${E_{p{k_1}}}({e_{min}}) \leftarrow \prod\nolimits_{i = 0}^{\ell - 1} {E_{p{k_1}}}{({e_{min,i}})^{{2^{\ell - i - 1}}}}$;
– ${\rm CSP}_1^{(1)}$, ${\rm CSP}_2^{(1)}$, and ${\rm CSP}_1^{(2)}$ execute ${E_{p{k_2}}}({e_{min}}) \leftarrow {\rm ST}({E_{p{k_1}}}({e_{min}}))$;
c) ${\rm CSP}_1^{(u)}$ and ${\rm CSP}_2^{(u)}$;
** for***i* = 1 to *n***do**
${E_{p{k_u}}}\left(\lambda _i^{(u)}\right) \leftarrow {E_{p{k_u}}}\left(e_i^{(u)} - {e_{min}}\right)$;
${E_{p{k_u}}}\left(\alpha _i^{(u)}\right) \leftarrow {E_{p{k_u}}}{\left(\lambda _i^{(u)}\right)^{r_i^{(u)}}}$, where $r_i^{(u)}{\ \in _R}\ {Z_N}$;
$\left[\alpha _i^{(u)}\right] \leftarrow {\rm SBD}\left({E_{p{k_u}}}\left(\alpha _i^{(u)}\right)\right)$;
${E_{p{k_u}}}\left(\beta _i^{(u)}\right) \leftarrow {\rm SBAOR}\left(\left[\alpha _i^{(u)}\right],[1]\right)$;
d) ${\rm CSP}_1^{(u)}$ and ${\rm CSP}_2^{(u)}$;
** for***i* = 1 to *n* and *j* = 1 to *m***do**
$\beta _{i,j}^{\prime (u)} \leftarrow {\rm SM}\left({E_{p{k_u}}}\left(\beta _i^{(u)}\right),{E_{p{k_u}}}\left(d_{i,j}^{(u)}\right)\right)$;
${E_{p{k_u}}}\left(d_{p,j}^{\prime(u)}\right) \leftarrow \prod\nolimits_{i = 1}^n \beta _{i,j}^{\prime (u)}$
e) ${\rm CSP}_1^{(2)}$, ${\rm CSP}_2^{(2)}$, and ${\rm CSP}_1^{(1)}$;
– ${E_{p{k_1}}}\left(d_p^{\prime (2)}\right) \leftarrow {\rm ST} \left({E_{p{k_2}}}\left(d_p^{\prime (2)}\right)\right)$;
f) ${\rm CSP}_1^{(1)}$;
– ${E_{p{k_1}}}({d_{mi{n_p}}}) \leftarrow {E_{p{k_1}}}\left(d_p^{\prime (1)} + d_p^{\prime(2)}\right) \leftarrow {E_{p{k_1}}}\left(d_1^{\prime (1)}\right)*{E_{p{k_1}}}\left(d_1^{\prime (2)}\right)$;
– ${E_{p{k_1}}}({S_p}) \leftarrow {E_{p{k_1}}}({S_{p - 1}})*{E_{p{k_1}}}({d_{mi{n_p}}})$;
g) ${\rm CSP}_1^{(u)}$ and ${\rm CSP}_2^{(u)}$;
** for***i* = 1 to *n* and *h* = 1 to $\ell$**do**
${E_{p{k_u}}}\left(e_{i,h}^{(u)}\right) \leftarrow {\rm SBOR}\left({E_{p{k_u}}}\left(\beta _i^{(u)}\right),{E_{p{k_u}}}\left(e_{i,h}^{(u)}\right)\right)$
4. ${\rm CSP}_1^{(1)}$;
** for***j* = 1 to *m***do**
– ${\gamma _j} \leftarrow {E_{p{k_1}}}({S_{k,j}}) \times {E_{p{k_1}}}({r_j})$, where *r*_*j*_ ∈_*R*_*Z*_*N*_;
– sends *γ*_*j*_ to CSP $_2^{(1)}$ and *r*_*j*_ to QO
5. ${\rm CSP}_2^{(1)}$;
** for***j* = 1 to *m***do**
– $\gamma _j^{\prime} \leftarrow {D_{s{k_1}}}({E_{p{k_1}}}({\gamma _j}))$;
– sends $\gamma _j^{\prime}$ to QO
6. QO;
a) **for***j* = 1 to *m***do**
$S_{k,j}^{\prime} \leftarrow \gamma _j^{\prime} - {r_j}$
b) computes the interpolation as $T = \langle S_{k,1}^{'}/k, \ldots ,S_{k,j}^{'}/k\rangle$;

At the second step of Algorithm 3, the servers ${\rm CSP}_1^{(u)}$ and ${\rm CSP}_2^{(u)}$ execute the protocols SSED and SBD. As stated in Elmehdwi, Samanthula & Jiang (2014), the outputs of the protocols will be in the encrypted format, and will only be revealed to the servers ${\rm CSP}_1^{(u)}$. Besides, no information about the plaintexts is revealed to any party during these protocols. At the step 3(a) of each iteration in Algorithm 3, the output of the protocol SMIN_*n*_ is only revealed to the servers ${\rm CSP}_1^{(u)}$. Besides, the SMIN_*n*_ protocol guarantees that the servers involved in the protocol do not know which records from both databases correspond to the current minimum distances. Similarly, the output of the SMIN protocol executed at the step 3(b) of Algorithm 3 is only revealed to the server ${\rm CSP}_1^{(1)}$. Also, the protocol does not reveal which record corresponds to the current global minimum.

The servers also run the ST protocol at the steps 3(b) and 3(e) of Algorithm 3 to transform the encryption of the current minimum distance under the public key $pk_{u_1}$ to the encryption under the public key $pk_{u_2}$. As we explained at the beginning of this section, the ST protocol protects the content of the encryption from all parties involved in the protocol. Furthermore, at the step 3(c), each server ${\rm CSP}_1^{(u)}$ runs the SBAOR protocol with ${\rm CSP}_2^{(u)}$ that outputs either the encryption of 1 just for the index corresponding to the current global minimum or the encryption of 0 for all the other indexes. Note that the SBAOR protocol uses the protocols SM and SBD as sub procedures, and it does not leak the index that corresponds to the current global minimum. Thus, data access patterns are protected from all the involved servers through the protocol, *i.e*. the servers do not know which data records used in producing the interpolation of *k*-nearest neighbors.

In conclusion, the STPkNN protocol preserves the confidentiality of the data, secures the privacy of user’s query point, and hides data access patterns.

### Complexity analysis

In this section, we will discuss the computation complexity of our protocol. The servers perform *n* instantiations of SSED and SBD protocols at the second step of the protocol. Since the computation complexity of the SSED protocol proposed in Elmehdwi, Samanthula & Jiang (2014) is bounded by *O*(*m*) multiplications and *O*(*m*) exponentiations, and the computation complexity of the SBD protocol proposed in Samanthula, Hu & Jiang (2013) is bounded by $O(\ell)$ multiplications and $O(\ell)$ exponentiations, the computation complexity of this step is bounded by $O(n \cdot (m + \ell))$ multiplications and $O(n \cdot (m + \ell))$ exponentiations.

On the other hand, at the third step of our protocol, the servers perform the following operations *O*(*k*) times: a single instantiation of SMIN_*n*_ protocol, a single instantiation of SMIN, 2 instantiations of ST protocol, *n* instantiations of SBD and SBAOR protocols, *n* · *m* instantiations of SM protocol, and $n \cdot \ell$ instantiations of SBOR protocol. The computation complexity of the SMIN_*n*_ protocol presented in this paper is bounded by $O(\ell \cdot n)$ multiplications and $O(\ell \cdot n)$ exponentiations and the computation complexity of the SMIN protocol presented in Elmehdwi, Samanthula & Jiang (2014) is bounded by $O(\ell)$ multiplications and $O(\ell)$ exponentiations. Besides, the ST protocol proposed in this paper, the SM protocol presented in Elmehdwi, Samanthula & Jiang (2014), and the SBOR protocol presented in Elmehdwi, Samanthula & Jiang (2014) only contain a constant number of multiplications and a constant number of exponentiations. Also, as we emphasized above, the computation complexity of the SBD protocol is bounded by $O(\ell)$ multiplications and $O(\ell)$ exponentiations (Samanthula, Hu & Jiang, 2013). Moreover, since the SBAOR protocol proposed in this paper deploys $\ell$ instantiations of SM protocol and $\ell\ -\ 1$ instantiations of SBOR protocols as sub procedures, the computation complexity of the SBAOR protocol bounded by by $O(\ell)$ multiplications and $O(\ell)$ exponentiations. Thus, the computation complexity of the third step is bounded by $O(k \cdot n \cdot (m + \ell))$ multiplications and exponentiations at total.

In addition, the servers perform only *O*(*m*) operations at the remaining steps of the protocol. Thus, the total computation complexity of our protocol is bounded by $O(k \cdot n \cdot (m + \ell))$ multiplications and exponentiations.

## Performance evaluation

In this section, we evaluated the performance of the proposed protocol STPkNN by carrying out a number of experiments under different parameter settings. We deployed Paillier cryptosystem (Paillier, 1999) for the encryption, and implemented the proposed protocols in Java. All the experiments were performed on a virtual Linux machine with an IntelR XeonR Two-CoreTM CPU 2.20 GHz processor and 4 GB RAM running Ubuntu 16.04 LTS. For the experiments, we utilized two real data sets from UCI machine learning repository (Dua & Graff, 2017); Heart Disease that consists of 600 data records such that each one contains 14 attributes concerning heart disease diagnosis, and Bank Marketing that contains 800 data records such that each one includes 15 attributes that helps to predict whether a new client will pay a term deposit. We first processed these data sets so that they contain only non-negative integer values. We then split each data set into two equal parts so that each one will be operated by a single cloud pair. Note that, for all the measurements, the experiment was repeated for multiple query points and the average time taken to execute a query was reflected to the table.

**Figure 3:**
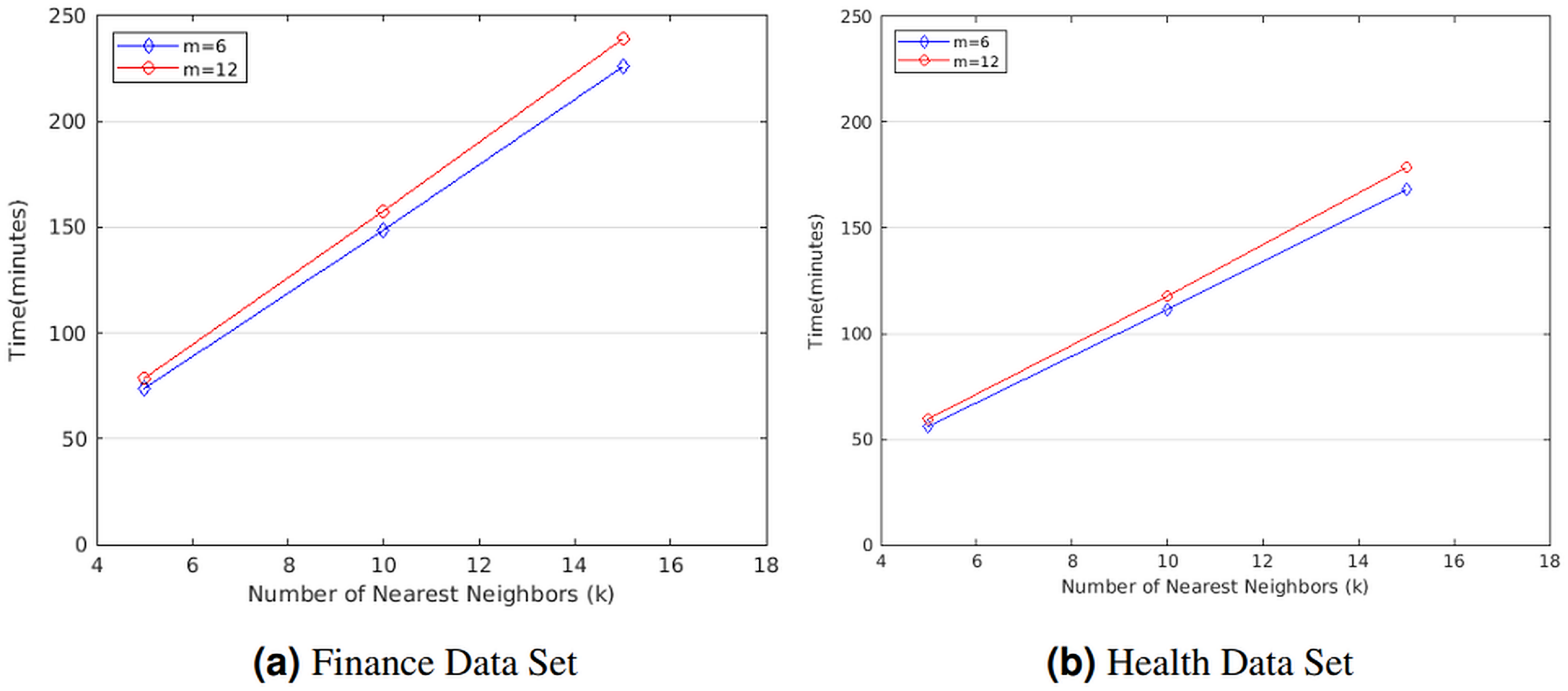
Running time of STPkNN for varying *k* values on the (A) finance and the (B) health data set.

We first evaluated the computation cost of STPkNN on finance data set in minutes for varying the number of nearest neighbors (*k*) and the number of attributes (*m*). As shown in Fig. 3A, if we fix the number of attributes as *m* = 6, the running time of our protocol varies from 74.08 to 226.16 min for finance data set when *k* is changed from 5 to 15. Besides, for *m* = 12, the running time of our protocol varies from 78.85 to 239.21 min when *k* is changed from 5 to 15. So the running time of our protocol grows linear with *k*. Also, we observe that the computation cost of our protocol increases by nearly a factor of 1.06 when *m* is doubled.

Similarly, we also evaluated the computation cost of STPkNN on heart disease data set in minutes for varying the number of nearest neighbors (*k*) and the number of attributes (*m*). As shown in Fig. 3B, if we set the number of attributes as *m* = 6, the running time of our protocol varies from 55.89 to 168.26 min when *k* is changed from 5 to 15. Besides, for *m* = 12, the running time of our protocol varies from 59.56 to 178.63 min when *k* is changed from 5 to 15. Thus, it is easy to observe that our protocol scales linearly with *k*. On the other hand, the running time of our protocol increases by almost a factor of 1.34 when the number of data records (n) is changed from 300 to 400. Thus, the running time of our protocol grows linear with n.

## Conclusions

In this study, we proposed a secure *k*-NN method that produces an interpolation of *k*-nearest neighbors to a query point over encrypted databases. We here claimed that instead of using one, employing two different databases in the protocol will yield more accurate and reliable interpolation value. We validated this claim by conducting experiments on publicly available real data sets. We also showed that our protocol preserves the confidentiality of data, assures the privacy of user’s query point, and hides data access patterns. We finally analyzed the performance of the proposed protocol through a number of experiments under different parameter settings. As a future study, we will examine and expand our work to apply other interpolation methods on encrypted data in distributed architecture. We will extend our protocol, that considers two encrypted databases stored in two different clouds, to multi-cloud settings.

## Supplemental Information

10.7717/peerj-cs.965/supp-1 Supplemental Information 1 Java Test Code.

10.7717/peerj-cs.965/supp-2 Supplemental Information 2 Java Test Code.

10.7717/peerj-cs.965/supp-3 Supplemental Information 3 Processed Finance Data.

10.7717/peerj-cs.965/supp-4 Supplemental Information 4 Processed Health Data.

## Additional Information and Declarations

### Data Availability

The following information was supplied regarding data availability: The data used in our experiments are available in the Supplemental Files. The data was taken from the UCI machine learning repository (Dua & Graff, 2017) and was processed before the experiments.
